# Combined Genotoxic Effects of a Polycyclic Aromatic Hydrocarbon (B(*a*)P) and an Heterocyclic Amine (PhIP) in Relation to Colorectal Carcinogenesis

**DOI:** 10.1371/journal.pone.0058591

**Published:** 2013-03-06

**Authors:** Emilien L. Jamin, Anne Riu, Thierry Douki, Laurent Debrauwer, Jean-Pierre Cravedi, Daniel Zalko, Marc Audebert

**Affiliations:** 1 INRA, UMR1331, Toxalim, Research Centre in Food Toxicology, Toulouse, France; 2 Université de Toulouse, INPT, UPS, UMR1331, Toulouse, France; 3 Laboratoire « Lésions des Acides Nucléiques », Université Joseph Fourier – Grenoble 1/CEA/Institut Nanoscience et Cryogénie/SCIB, UMR-E3, Grenoble, France; University Paris Diderot-Paris 7, France

## Abstract

Colorectal neoplasia is the third most common cancer worldwide. Environmental factors such as diet are known to be involved in the etiology of this cancer. Several epidemiological studies have suggested that specific neo-formed mutagenic compounds related to meat consumption are an underlying factor involved in the association between diet and colorectal cancer. Heterocyclic amines (HCAs) and polycyclic aromatic hydrocarbons (PAHs) are known mutagens and possible human carcinogens formed at the same time in meat during cooking processes. We studied the genotoxicity of the model PAH benzo(*a*)pyrene (B(*a*)P) and HCA 2-amino-1-methyl-6-phenylimidazo[4,5-*b*]pyridine (PhIP), alone or in mixture, using the mouse intestinal cell line Apc^Min/+^, mimicking the early step of colorectal carcinogenesis, and control Apc^+/+^ cells. The genotoxicity of B(*a*)P and PhIP was investigated using both cell lines, through the quantification of B(*a*)P and PhIP derived DNA adducts, as well as the use of a genotoxic assay based on histone H2AX phosphorylation quantification. Our results demonstrate that heterozygous *Apc* mutated cells are more effective to metabolize B(*a*)P. We also established in different experiments that PhIP and B(*a*)P were more genotoxic on Apc*^Min/+^* cells compared to Apc*^+/+^*. Moreover when tested in mixture, we observed a combined genotoxicity of B(*a*)P and PhIP on the two cell lines, with an increase of PhIP derived DNA adducts in the presence of B(*a*)P. Because of their genotoxic effects observed on heterozygous *Apc* mutated cells and their possible combined genotoxic effects, both B(*a)*P and PhIP, taken together, could be implicated in the observed association between meat consumption and colorectal cancer.

## Introduction

Colorectal cancer is one of the most common cancers in the western world [1]. The implication of dietary habits in colorectal carcinogenesis is suggested by several epidemiological studies [2], [3], with a possible link between colorectal carcinogenesis and the presence in the diet of heterocyclic amines (HCAs) and polycyclic aromatic hydrocarbons (PAHs) [4], [5], [6]. Moreover, PAHs and HCAs have been shown to induce tumors in different animal models [7], [8], [9].

Colon cancer is described as a multistep process [10] in which mutation in the *adenomatous polyposis coli* (*Apc*) gene is considered to be the earliest event in the initiation of colorectal carcinogenesis [11]. Min mice (Apc^Min/+^), which retain the heterozygous *Apc* genotype have been shown to be more sensitive to the carcinogenic potential of 2-amino-1-methyl-6-phenylimidazo[4,5-*b*]pyridine (PhIP, an extensively studied HCA) than control C57BL/6J mice (Apc^+/+^) [12]. Several *in vitro* studies have been published regarding the characterization of tumor-promoting factors for colon carcinogenesis. In such studies, the cellular model used plays a crucial role in understanding the relationship between mutagenic compounds and the etiology of cancer. Up to now, *in vitro* studies on colorectal carcinogenesis have usually been performed on carcinoma cell lines, which are not the best suited to investigate the biological effect of xenobiotics since normal or premalignant epithelial cells are the primary targets of these compounds [13]. Intestinal cell lines derived from C57BL/6J mice (Apc^+/+^) and Min mice (Apc^Min/+^) which retains the heterozygous *Apc* genotype, have been developed [14], [15], [16]. This cellular model can contribute to gain a better understanding of the biological effects of promoting compounds on normal (Apc^+/+^) or premalignant cells (Apc^Min/+^) [17]. PAHs, like HCAs, require metabolic bioactivation to express their carcinogenic potential through specific DNA-adducts formation [18], [19], [20]. Apc^Min/+^ cells have been reported to have a greater potency in the bioactivation of compounds such as oestradiol or PhIP compared to Apc^+/+^ cells [21], [22]. Based on these data, the promoting effect of PhIP could be explained by a higher probability to generate new *in situ* mutations [21]. Moreover, different studies in human and rodent have demonstrated that PhIP is bioavailable to the colon and forms protein and DNA adducts in a dose-dependent manner [23], [24], [25].

In the present study, we examined the genotoxicity of two well-known genotoxicants and carcinogens, namely B(*a*)P and PhIP. These models PAH and HCA were tested alone or in mixture, using the mouse intestinal cell lines Apc^+/+^ and Apc^Min/+^. Since both compounds are pro-carcinogens, and that the PhIP metabolic profiles produced by the two cell lines were previously reported to be different [22], we also investigated the bioactivation of B(*a*)P on Apc^+/+^ and Apc^Min/+^ cell lines.

The genotoxicity, and subsequently the carcinogenicity of PhIP and B(*a*)P are directly linked to the formation of dG-C8-PhIP- and dG-N^2^-BPDE-DNA adducts, respectively [18], [19], [20], [26]. In order to evaluate the genotoxic potential of these two compounds, alone or in mixture on the two cell lines, we quantified B(*a*)P and PhIP derived DNA adducts by HPLC-MS/MS. Moreover, we used a genotoxic assay based on the quantification of the phosphorylation of the histone H2AX (named γH2AX) that reflects a global genotoxic insult resulting from diverse types of DNA damages, notably DNA adducts and oxidative DNA lesions [27], [28], [29], [30], [31], [32], [33], [34].

## Materials and Methods

Caution: B(a)P and PhIP are carcinogens and potentially tumorigenic to humans. These compounds should be handled with care (NIH Guideline for the Use of Chemical Carcinogens).

### Chemicals and Reagents

[^14^C]-benzo(*a)*pyrene (radio-HPLC purity >97.5%; specific activity: 2 GBq/mmol) was supplied by Amersham (Brea, CA). Benzo(*a*)pyrene and dimethyl sulfoxide (DMSO) (with chemical purity >99%) were obtained from Sigma-Aldrich (Saint Quentin Fallavier, France). 2-Amino-1-methyl-6-phenylimidazo[4,5-b]pyridine (PhIP) as well as dG-C^8^-PhIP adducts were purchased from Toronto Research Chemicals (Ontario, Canada). Cells were exposed to 0.2% (v/v) DMSO in culture medium.

Flo-Scint II and Ultima Gold liquid scintillation cocktails were purchased from PerkinElmer Life and Analytical Sciences (Courtaboeuf, France). HPLC-grade solvents were purchased from Scharlau (Barcelona, Spain). Water used for HPLC analyses was purified with a Milli-Q system (Millipore, Saint-Quentin-en-Yvelines, France). Alkaline phosphatase, RNase A and RNase T1 were purchased from Roche Applied Science (Meylan, France). Phosphodiesterases I and II, Nuclease P1, Tris-HCl, succinic acid, CaCl_2_, sucrose, NaI, EDTA, MgCl_2_, deferoxamine, Triton X100 and bovine serum albumin (BSA) were obtained from Sigma-Aldrich (Saint Quentin Fallavier, France). Proteinase K came from Qiagen (Courtaboeuf, France) and SDS from Euromedex (Souffelweyersheim France). ZnSO_4_ and (NH_4_)_2_SO_4_ were purchased from Merck Millipore (Fontenay-sous-Bois, France).

### Cell Lines and Cultures

Apc^+/+^ and Apc^Min/+^ have been previously described [14], [15], [21], [22], [35]. Cells were cultured in Dulbecco-modified essential medium (DMEM) supplemented with 10% (v/v) fetal calf sera, 1% (v/v) penicillin/streptomycin, and 10 U/ml interferon γ. The Apc*^Min/+^* and Apc*^+/+^* cell lines were a generous gift from Dr F. Pierre (INRA, UMR1331, Toxalim, Toulouse, France).

### Benzo(*a*)pyrene Metabolic Profiling

For each cell line (Apc^+/+^ or Apc^Min/+^), 3×10^5^ cells per well were grown at 33°C in 95% air and 5% CO_2_ in 12 wells plates containing 1 mL medium per well. After 16 h, the medium was replaced by a serum free medium and 2 µL [^14^C]-B(*a*)P (2250 Bq per well, corresponding to 3 µM) diluted in DMSO were added as described previously [27], [28]. At the end of the incubation period (24 h), culture media were removed and stored at –20°C until analysis. Cells were recovered by washing each well with 1 mL water/acetonitrile (50/50, v/v), stored at -20°C until analysis. Radioactivity contained in supernatant and cells (10 µL aliquots) was quantified by direct counting in a Packard scintillation counter (Model Tri-Carb 200CA; PerkinElmer) using Ultima Gold as the scintillation cocktail. More than 95% of the radioactivity was recovered at the end of each experiment. For all vials, sample quenching was compensated by the use of quench curves and external standardization. Experiments were carried out in triplicate.

For B(*a*)P metabolic profiling, the radio-HPLC system was as previously described [28], [36]. In such conditions, B(*a*)P retention time (R*_T_*) was 52 min. Metabolites were quantified by integrating the area under peaks monitored by radioactivity detection. Identification of B(*a*)P metabolites was achieved by liquid chromatography coupled to mass spectrometry (HPLC-MS/MS) as previously reported [28], [36].

### DNA Extraction and Digestion

For each cell line (Apc^+/+^ or Apc^Min/+^), 10×10^6^ cells were grown at 33°C in 95% air and 5% CO_2_ in a 100 mm petri dish containing 10 mL medium per well. After 16 h, the medium was replaced by 5 mL medium without serum and PhIP and/or B(*a*)P diluted in DMSO were added. Experiments were carried out in triplicate. Then, DNA was extracted by a two-step protocol as previously described [37]. Briefly, the nuclei were extracted by centrifugation of the cellular pellet suspended in a lysis buffer A (320 mM sucrose, 5 mM MgCl_2_, 10 mM Tris-HCl, 0.1 mM deferoxamine 1% Triton X100, pH 7.5). Lysis of the nuclear membrane was performed by agitation of extracted nuclei dissolved in a lysis buffer B (10 mM Tris-HCl, 5 mM EDTA, 0.15 mM deferoxamine, pH 8.0) and SDS 10%. RNAse A and RNAse T1 were added and samples were incubated 15 min at 50°C. Proteinase K was added prior to incubation at 37°C for 1 h to eliminate proteins. DNA precipitation was performed by adding a NaI solution (7.6 M NaI, 40 mM Tris-HCl, 20 mM EDTA, 0.3 mM deferoxamine, pH 8.0) and 2-propanol. DNA was then extracted by successive centrifugations and washings by 2-propanol (40%) and ethanol (70%). Finally, DNA was recovered in 0.1 mM deferoxamine.

For each sample, 50 µg of DNA quantified using a Nano-drop system (Thermo Scientific, Les Ullis, France) was digested to nucleosides as follows. Phosphodiesterase II, Nuclease P1 and P1 buffer 10X (200 mM succinic acid, 100 mM CaCl_2_, pH 6.0) were added to DNA samples. Incubation was performed for 2 h at 37°C. Then, alkaline phosphatase, phosphodiesterase I and Palk buffer 10X (500 mM Tris-HCl, 1 mM EDTA, pH 8.5) were added and samples were incubated for 2 h at 37°C. Finally, 10 µL of 0.1 M HCl were added to neutralize the solution.

### DNA Adducts Quantification

DNA adducts were quantified as modified deoxynucleosides. The digested mixture was then evaporated to dryness and dissolved in H_2_O/CH_3_OH 1/1 (v/v) prior injection on a HPLC-tandem mass spectrometry (HPLC-MS/MS) system. The HPLC-MS/MS system used for the dG-N^2^-BPDE DNA adducts quantification consisted first in an API 3000 mass spectrometer (SCIEX) using the multiple reaction monitoring mode with positive electrospray ionization. The HPLC-MS/MS method was developed thanks to the preparation of pure and calibrated solutions of authentic dG-N^2^-BPDE [38]. The monitored fragmentations for the diol epoxide adduct of B(*a*)P were: *m/z* 570 [M+H]^+^ → *m/z* 454 [M+H – 116]^+^; *m/z* 570 [M+H]^+^ → *m/z* 257 [BPDE+H – H_2_O – CO]^+^; *m/z* 570 [M+H]^+^ → *m/z* 285 [BPDE+H – CO]^+^. Chromatographic separations were achieved using an octadecylsilyl reversed phase Uptisphere ODB column (2×150 mm, 5 µm particle size, Montluçon, France). The samples were suspended in a mixture of H_2_O/CH_3_OH 1/1 (v/v). The elution was performed using a linear gradient from 0% to 100% of acetonitrile in 2 mM ammonium formate over 30 min at a flow rate of 0.2 ml/min. The retention time of dG-N^2^-BPDE was 21 minutes. The amounts of unmodified nucleosides were quantified using a UV detection (λ = 270 nm). An external calibration, based on the injection of known amounts of analyte, was used to quantify the respective amounts of adducts and normal nucleosides. The simultaneous measurement of adducts and normal nucleosides in a same sample allowed to compensate for possible errors in injection volume. In addition, the large difference in the respective retention times of normal nucleosides versus adducts, prevented matrix effect of the MS/MS detection. Last, the straightforward sample treatment without prepurification avoided loss of material. Altogether, no internal standard was used.

The dG-C8-PhIP adducts were quantified by HPLC-MS/MS using of a Surveyor HPLC pump (Thermo Scientific, Les Ullis, France) associated with a TSQ Quantum Discovery Max triple quadrupole (Thermo Scientific, Les Ullis, France). Chromatography was achieved with a XTerra C18 column (2.1×150 mm, 3.5 µm particle size, Waters, Saint Quentin en Yvelines, France). Mobile phases were eluted at a flow rate of 0.2 mL/min at 22°C and were composed of A: H_2_O/CH_3_OH/CH_3_CO_2_H 90/10/0.2 (v/v/v) and B: H_2_O/MeOH/CH_3_CO_2_H 10/90/0.2 (v/v/v). The elution program was: 0 min 0% of B, from 4 min to 11 min 67% of B, from 12 min to 17 min 100% of B and from 18 min to 23 min 0% of B. A volume of 10 µL of sample was injected. The HPLC system was coupled to the mass spectrometer equipped with an electrospray ionization source. DNA adduct quantification was performed in the positive ionization mode using the selected reaction monitoring detection method with the transition *m/z* 490 [M+H]^+^ → *m/z* 374 [M+H - 116]^+^. Optimized electrospray ionization conditions were as follow: spray voltage 4500 V, sheath gas (N_2_) 12 au, auxiliary gas (N_2_) 25 au, capillary temperature 370°C, capillary potential 35 V. PhIP adducts were quantified by external calibration using commercial dG-C8-PhIP adducts at concentrations of 0.15, 0.3, 0.75, 1.5 and 3 ng/mL, injected in triplicate. The performances of the dG-C8-PhIP quantification method by HPLC-MS/MS corresponded to: linearity R^2^>0.998, intra-day variability RSD <4% (*n* = 3), accuracy >93% (measured at 0.2, 1 and 2 ng/mL *n* = 3) and no carry-over effect. The external calibration was validated by quantifying dG-C8-PhIP adducts in blank samples spiked (1.5 ng/mL) before and after the digestion protocol (*n* = 3). A yield of 114% (±9%) was found in samples spiked before and 96% (±7%) in samples spiked after the digestion protocol, showing no matrix effect as well as no loss of adduct during the sample preparation. The limit of detection (signal intensity/noise intensity (S/N) >3) and the limit of quantification (S/N>10) were estimated from real samples at a level of 3 dG-C8-PhIP adducts/10^8^ normal nucleosides and 10 adducts/10^8^ normal nucleosides, respectively.

### H2AX In Cell Western (ICW) Assay

The H2AX In Cell Western technique was performed as previously described with the following modifications [27], [28], [29], [30], [34]. Briefly, cells were dispensed in 96-well cell culture plate (40×10^3^ cells, 200 µL/well) and were treated in duplicate, 16 h later, with the model compounds or vehicle (DMSO) in serum free medium. After 24 h treatment, cells were washed in PBS and directly fixed in the plate with 4% paraformaldehyde (Electron Microscopy Science) in PBS for 20 min at room temperature (RT), then washed using PBS for 5 min. Paraformaldehyde was neutralized with 20 mM NH_4_Cl for 2 min and then the samples were washed with PBS for 5 min. Cells were permeabilized with 0.2% Triton X-100 in PBS for 5 min and washed with PBS, 2% fetal calf serum, 0.2% Triton X-100 (PST buffer). Cells were blocked with MAXblock Blocking medium (Active Motif, Belgium) supplemented with phosphatase inhibitor PHOSTOP (Roche) for 60 min at RT, followed by 2 h incubation with rabbit monoclonal anti γH2AX (Cell Signaling) in PST buffer. After three 5 min washes in PST, secondary detection was carried out using an infrared fluorescent dye conjugated to goat antibody with an absorption peak at 770 nm (CF770, Biotium) in PST buffer. For DNA labeling, TO-PRO-3 iodide (Molecular Probes) in PST was used in conjugation with the secondary antibody. After 1 h incubation and three 5 min washes in PST, DNA and γH2AX were simultaneously visualized using an Odyssey Infrared Imaging Scanner (Li-CorScienceTec, Les Ulis, France) with the 700-nm fluorophore (red color) and the 800-nm fluorophore (green dye), respectively. Raw absorbance data was averaged for the duplicate, corrected for background; the relative fluorescence units from the scanning allowed a quantitative analysis. The ICW technique allows the determination of cytotoxicity and genotoxicity in a single experiment [27], [28], [30], [34]. For determination of genotoxicity, relative fluorescent units for γH2AX per cell (as determined by γH2AX divided by DNA content) were divided by the respective controls (vehicle only), in order to determine the change in phosphorylation of H2AX levels relative to control. For determination of cytotoxicity, DNA content recorded in the different experiments was compared to DNA content in control cells. Statistical analysis was performed to assess significant effects of chemical treatments. All experiments were performed at least four times independently.

### Data Analysis

Statistical analyses were performed using Student’s *t*-test. Statistical analysis was performed using the R Software. Error bars represent SEM (the standard error of the mean). Statistically significant increase in H2AX phosphorylation compared with DMSO control using Student’s test; *, *p*<0.05; **, *p*<0.01. Statistically significant difference between Apc^+/+^ and Apc^Min/+^ cells using Student’s test; b, *p*<0.05; a, *p*<0.01.

## Results

### Benzo(*a*)pyrene Metabolism on Apc^+/+^ and Apc^Min/+^ Cell Lines

**Figure 1:**
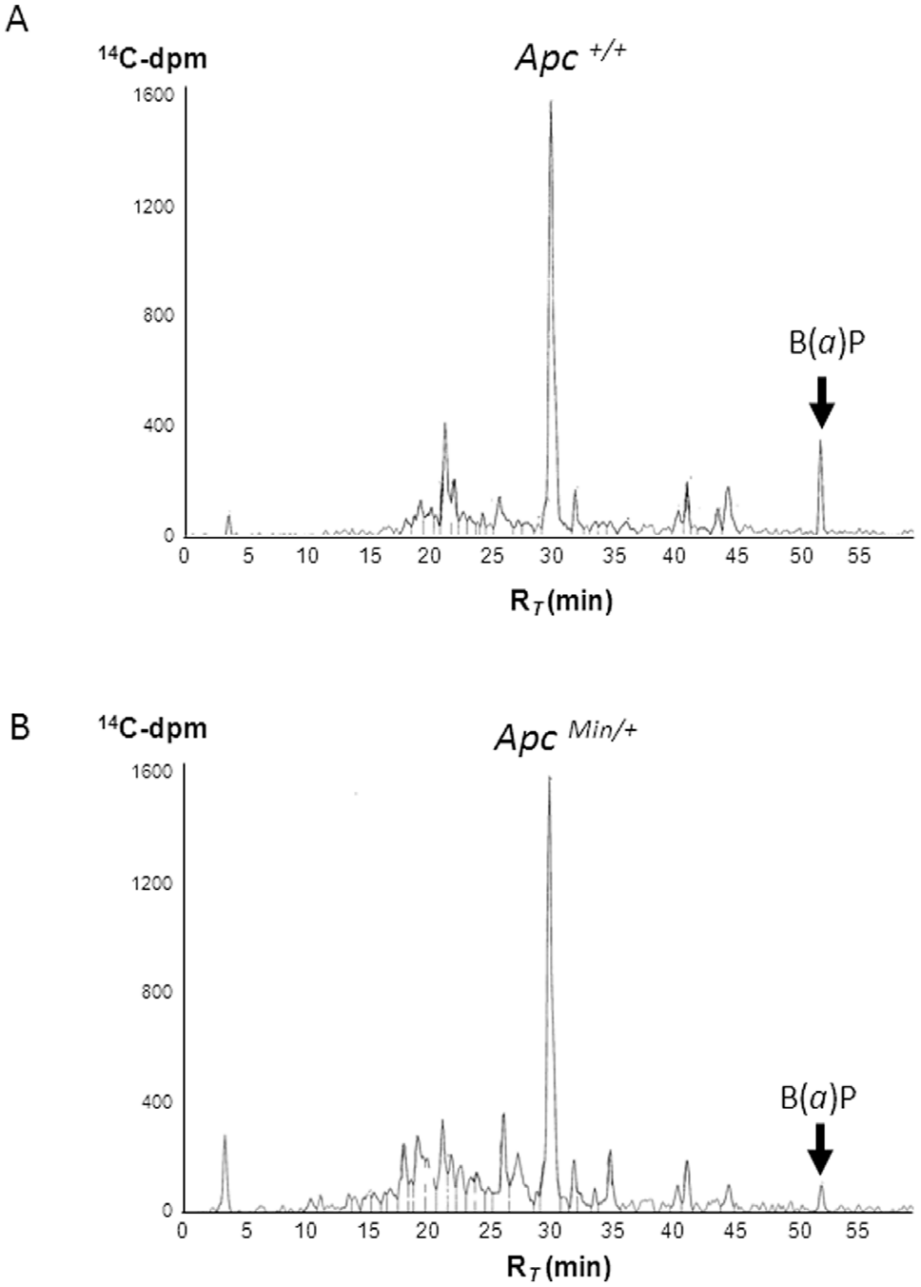
Metabolism of benzo(*a*)pyrene in Apc^+/+^ and Apc^Min/+^ cell lines. Typical radio-HPLC profiles of supernatants obtained from incubation of 3 µM [^14^C]-B(*a*)P with (A) Apc^+/+^ and (B) Apc^Min/+^ cell lines (incubation time: 24 h).

The genotoxicity of heterocyclic amines (HCAs) and polycyclic aromatic hydrocarbons (PAHs) mainly results from metabolic pathways leading to the formation of highly reactive intermediates responsible for the formation of DNA adducts [18], [19], [20], [26]. Previously, Apc^Min/+^ cells have been reported to have a greater potency than Apc^+/+^ cells, regarding the biotransformation of different compounds into bioactive metabolites [21], [22]. This was in particular well established for PhIP [21]. We investigated whether this difference between the two cell lines could also be observed for PAHs. For this purpose, we analyzed the biotransformation of [^14^C]-benzo(*a*)pyrene ([^14^C]-B(*a*)P) on Apc^+/+^ and Apc^Min/+^ cell lines (Fig. 1). After incubation of the two cell lines with [^14^C]-B(*a*)P for 24 h, both supernatants (culture media) and cell extracts were analyzed by radio-HPLC. In the supernatant of the incubation with no cells (control), more than 98% of the radioactivity was recovered as unchanged [^14^C]-B(*a*)P (data not shown). B(*a*)P was extensively metabolized by both Apc^+/+^ and Apc^Min/+^ cells. However, as observed in Fig. 1A and 1B, the profiles obtained for Apc*^+/+^* and Apc*^Min/+^* supernatants were slightly different, with a higher number of polar metabolites (retention times below 25 min) for Apc*^Min/+^* cells. Supernatants and cell extracts analyses (Table 1), indicated that B(*a*)P was metabolized at a higher extent by Apc^Min/+^ cells, as compared with Apc^+/+^ cells, with unchanged B(*a*)P representing 8.2% versus 28.7% of the total radioactivity, respectively. A major metabolite was observed for the two cell lines at R*_T_* = 30 min (Fig. 1A and 1B), and accounted for nearly 30% of the total radioactivity (Table 1). It was identified by HPLC-MS as a B(*a*)P-OH conjugated to glucuronic acid (BaP-OGlc) (Table 1). By comparison of their mass spectra and retention times with standard compounds [30], peaks detected at 40.0 min, 40.8 min and 44.1 min were identified as unconjugated B(*a*)P-9-OH, B(*a*)P-3-OH and B(*a*)P-7-OH, respectively (Table 1). Higher amount of unconjugated B(*a*)P-OH metabolites (17.7% versus 5.5%) was observed on Apc^+/+^ compared to Apc^Min/+^ cell line (Table 1).

**Table 1:** Production of B(*a*)P metabolites (supernatants and cell extracts) on Apc^+/+^ and Apc^Min/+^ cell lines after 24 h incubations with 3 µM [^14^C]-B(*a*)P.

Identified Metabolites	Apc^+/+^	Apc^Min/+^
BaP-OGlc	30.8±2.1	34.2±1.5
BaP-OSO_3_H	1.2±0.5	nd
BaP-OH	17.7±1.1^**^	5.5±0.5
BaP	28.7±2.4 ^**^	8.2±0.7
Unidentified metabolites	21.6±2.0	52.1±1.4

nd : not detected.

Values are expressed as a percentage of the total radioactivity and are means ± SD. Significant differences between Apc^+/+^ and Apc^Min/+^ cells were determined using Student’s test (**<0.01).

### B(*a*)P Genotoxicity on Apc^+/+^ and Apc^Min/+^ Cell Lines

**Figure 2:**
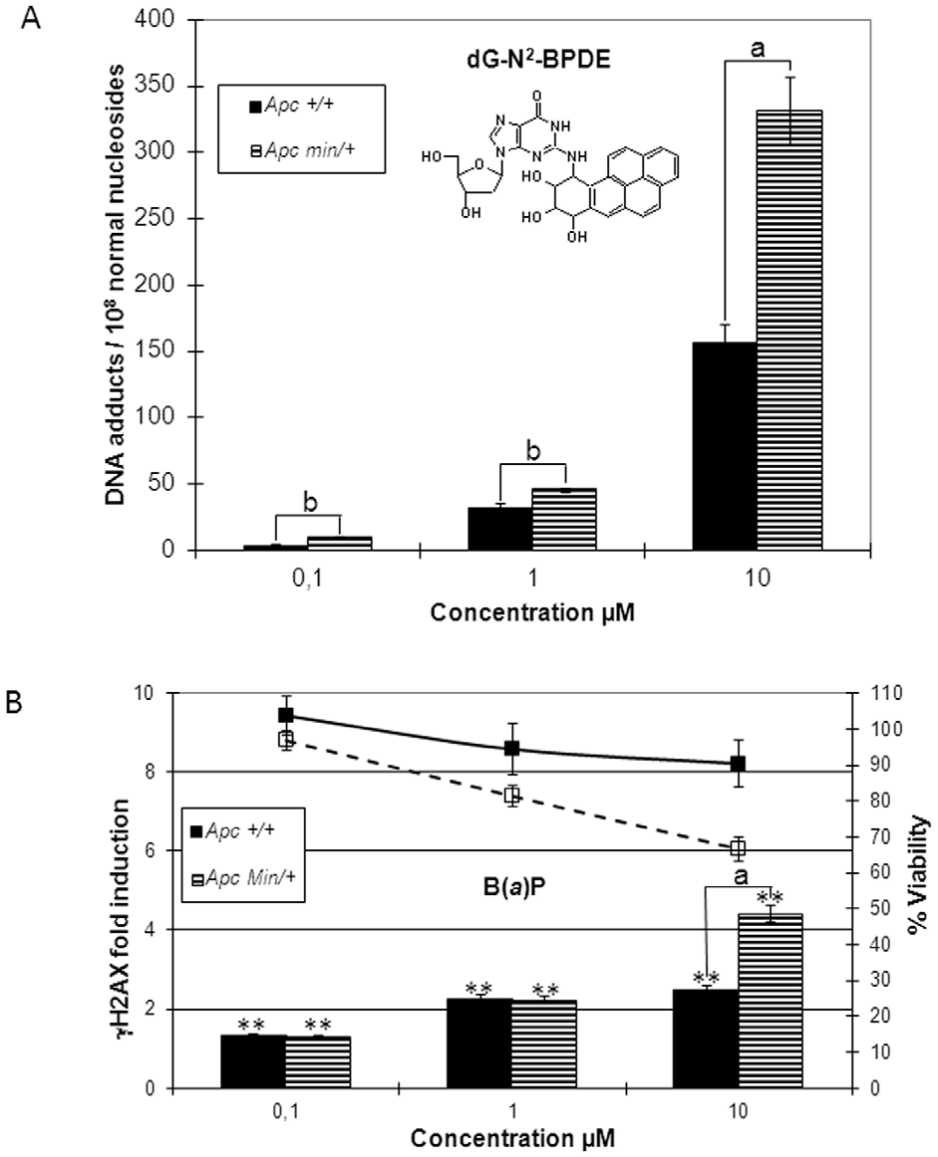
Genotoxicity of B(*a*)P in Apc^+/+^ and Apc^Min/+^ cell lines. Apc^+/+^ and Apc^Min/+^ cells were treated with the indicated concentrations of BaP for 24 h. (A) Genotoxicity was evaluated by HPLC-MS/MS quantification of dG-N^2^-BPDE in DNA. (B) Genotoxicity was evaluated with γHAX ICW assay. Bars represent the average of at least three independent experiments with SEM. Statistically significant increase in H2AX phosphorylation compared with DMSO control using Student’s test; *, *p*<0.05; **, *p*<0.01. Statistically significant difference between Apc^+/+^ and Apc^Min/+^ cells using Student’s test; b, *p*<0.05; a, *p*<0.01.

The genotoxicity of B(*a*)P on Apc^+/+^ and Apc^Min/+^ cells was assayed after 24 h treatment (Fig. 2). The adduct of the diol epoxide metabolite to guanine (dG-N^2^-BPDE), known to be the major DNA adduct of B(*a*)P, was quantified on the two cell lines using LC-MS/MS (Fig. 2A). At the three B(*a*)P concentrations tested (0.1, 1 and 10 µM), significantly more dG-N^2^-BPDE DNA adducts were formed in incubations carried out with the Apc^Min/+^ cell line, compared to the Apc^+/+^ cell line (Table 2). At the highest concentration tested (10 µM), dG-N^2^-BPDE DNA adduct was detected on Apc^Min/+^ cells at a mean level (*n* = 3) of 331 (±45) DNA adducts/10^8^ normal nucleosides, compared to only 156 (±24) DNA adducts/10^8^ normal nucleosides on Apc^+/+^ cells (Table 2). For both cell lines, a significant B(*a*)P dose-dependent increase in H2AX phosphorylation was found at all the concentrations tested (Fig. 2B). Moreover, at 10 µM of B(*a*)P, a clear difference between the two cell lines was also noticed for cytotoxicity (67% versus 90% viability) as well as for genotoxicity (4.25 versus 2.40 γH2AX fold induction) on Apc^Min/+^ compared to Apc^+/+^ cell line (Fig. 2B and Table 2).

**Table 2:** Genotoxicity and cytotoxicity of B(*a*)P and PhIP in Apc^+/+^ and Apc^Min/+^ cell lines.

Cell line	Apc^+/+^			Apc^Min/+^		
Treatment	DNAadducts/(10^8 ^nn)	γH2AX foldinduction	Viability%	DNAadducts/(10^8 ^nn)	γH2AX foldinduction	Viability%
PhIP 1 µM	nd	1.07 (0.14)	100 (1)	13 (3)	1.12 (0.11)	96 (2)
PhIP 3 µM	nt	1.11 (0.16)	104 (4)	nt	1.19 (0.08)	97 (2)
PhIP 10 µM	12 (1)	1.16 (0.12) ^b^	98 (4)	93 (20)	1.43 (0.17) ^**^	95 (1)
PhIP 30 µM	32 (6)	1.27 (0.19) *, b	97 (4)	561 (20)	2.11 (0.41) ^**^	81 (3)
PhIP 100 µM	169 (60)	1.53 (0.26) ^**, a^	88 (5)	6108 (1000)	2.68 (0.50) ^**^	67 (2)
BaP 0.1 µM	4 (2) ^b^	1.32 (0.11) ^**^	104 (5)	9 (1)	1.29 (0.07) ^**^	97 (3)
BaP 1 µM	32 (6) ^b^	2.18 (0.29) ^**^	94 (7)	45 (4)	2.41 (0.21) ^**^	81 (3)
BaP 10 µM	156 (24) ^a^	2.40 (0.33) ^**, a^	90 (7)	331 (45)	4.25 (0.56) ^**^	67 (4)
BaP 1 µM/PhIP10 µM	27 (1)/64 (10) ^a^	1.90 (0.30)	94 (5)	63 (13)/468 (23)	2.15 (0.25)	76 (2)

nn : normal nucleosides.

nd : not detected.

nt : not tested.

Values are expressed as means (SEM), n≥3.

* : Statistically significant increase in H2AX phosphorylation compared with DMSO control was determined using Student’s test (*, *p*<0.05; **, *p*<0.01); ^a, b^: significant difference between Apc^+/+^ and Apc^Min/+^ cells using Student’s test (a, *p*<0.05; b, *p*<0.01).

### PhIP Genotoxicity on Apc^+/+^ and Apc^Min/+^ Cell Lines

**Figure 3:**
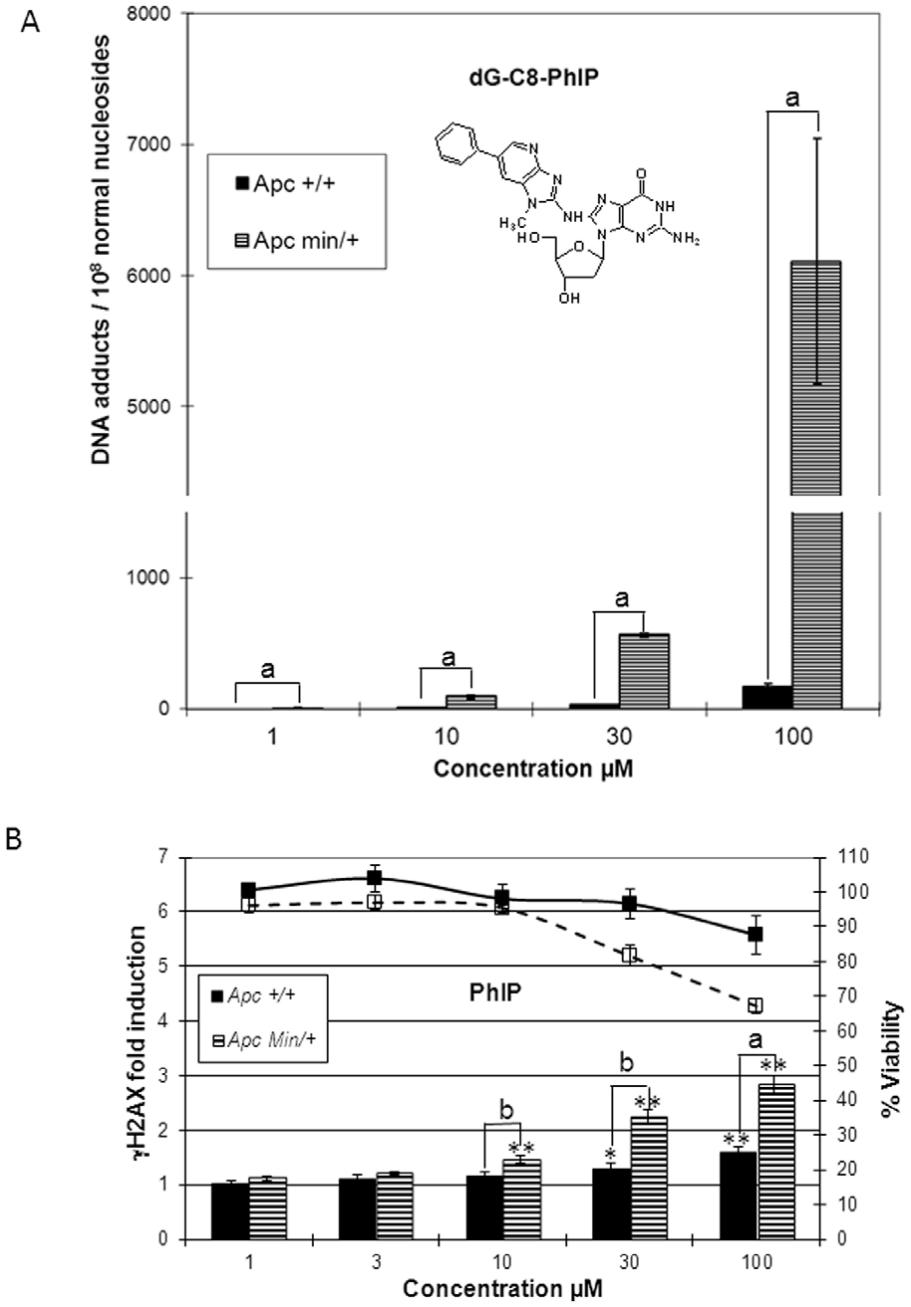
Genotoxicity of PhIP in Apc^+/+^ and Apc^Min/+^ cell lines. Apc^+/+^ and Apc^Min/+^ cells were treated with the indicated concentrations of PhIP for 24 h. (A) Genotoxicity was evaluated by HPLC-MS/MS quantification of dG-C8-PhIP in DNA. (B) Genotoxicity was evaluated with γHAX ICW assay. Bars represent the average of at least three independent experiments with SEM. Statistically significant increase in H2AX phosphorylation compared with DMSO control using Student’s test; *, *p*<0.05; **, *p*<0.01. Statistically significant difference between Apc^+/+^ and Apc^Min/+^ cells using Student’s test; b, *p*<0.05; a, *p*<0.01.

Apc^Min/+^ cells have been previously reported to be able to bio-activate PhIP more efficiently than their *Apc^+/+^* analog [21]. We thus analyzed the genotoxicity of PhIP on Apc^+/+^ and Apc^Min/+^ cells after 24 h treatment through the quantification on the two cells lines of dG-C8-PhIP, the well-known DNA adduct formed through the bioactivation of PhIP [39], [40]. Like for B(*a*)P, at the four PhIP concentrations tested (1, 10, 30 and 100 µM) more dG-C8-PhIP DNA adducts were produced on Apc^Min/+^ compared to Apc^+/+^ cell line (Fig. 3A and Table 2). A dose-dependent increase in the difference between the two cell lines was observed, with a 7 fold difference at 10 µM of PhIP, mean level (*n* = 3) of dG-C8-PhIP DNA adducts (12 (±1)/10^8^ normal nucleosides on Apc^+/+^ versus 93 (±20)/10^8^ normal nucleosides on Apc^Min/+^), a 17 fold difference at 30 µM (32 (±6)/10^8^ normal nucleosides versus 561 (±20)/10^8^ normal nucleosides) and a 36 fold difference at 100 µM (169 (±60)/10^8^ normal nucleosides versus 6108 (±1000)/10^8^ normal nucleosides).

As found with B(*a*)P (Fig. 2), the *APC* genotype genotoxic differential for PhIP was confirmed by the γH2AX assay (Fig. 3B). A significant PhIP dose-dependent increase in H2AX phosphorylation was observed on both cell lines. H2AX phosphorylation was found on the Apc^+/+^ cell line at 30 and 100 µM and from 10 µM on Apc^Min/+^ (Fig. 3B and Table 2). Moreover, at the three highest concentrations tested (10, 30 and 100 µM), like in the dG-C8-PhIP DNA adducts quantification (Fig. 3A), a clear difference between the two cell lines was observed. In particular, at 100 µM PhIP, higher cytotoxicity (67% versus 88% viability) and genotoxicity (2.68 versus 1.53 γH2AX fold induction) were noticed on Apc^Min/+^ cell line, compared to the Apc^+/+^ cell line (Fig. 3B and Table 2).

### Genotoxicity of PhIP and B(*a*)P in Mixture on Apc^+/+^ and Apc^Min/+^ Cell Lines

**Figure 4:**
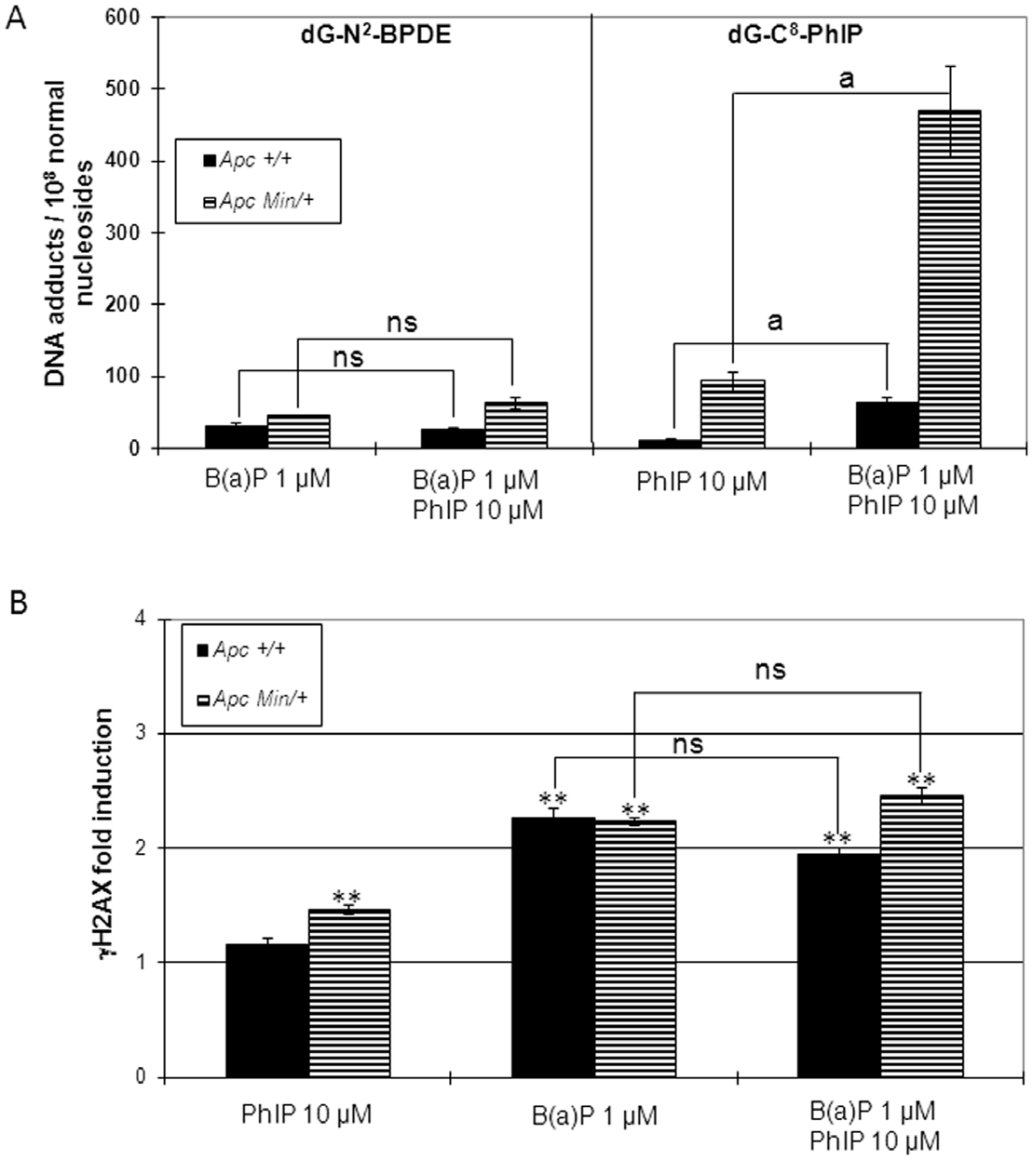
Synergic genotoxicity of B(*a*)P and PhIP in Apc^+/+^ and Apc^Min/+^ cell lines. Apc^+/+^ and Apc^Min/+^ cells were treated with the indicated concentrations of BaP and PhIP for 24 h. (A) Genotoxicity was evaluated by quantification by HPLC-MS/MS quantification of B(*a*)P- and PhIP-derived DNA adducts. (B) Genotoxicity was evaluated with γHAX ICW assay. Bars represent the average of at least three independent experiments with SEM. Statistically significant increase in H2AX phosphorylation compared with DMSO control using Student’s test; **, *p*<0.01. Statistically difference between single and combined treatment using Student’s test; ns, not significant; a, *p*<0.01.

Previously, pre-treatment of the cell lines used with 2,3,7,8-tetrachlorodibenzo-*p*-dioxin (TCDD), an inducer of the cytochromes P450 of the CYP1 family, has been reported to induce the bioactivation of different compounds [21], [22], notably PhIP [21]. Because B(*a*)P has been shown to be also a CYP1 family inducer [41], we analyzed the genotoxicity of a mixture of B(*a*)P (1µM) and PhIP (10 µM) on Apc^+/+^ and Apc^Min/+^ cells after 24 h treatment (Fig. 4). dG-C8-PhIP- and dG-N^2^-BPDE-DNA adducts were quantified in parallel on the two cells lines (Fig. 4A). No significant difference in dG-N^2^-BPDE-DNA adducts formation was observed after treatment with B(*a*)P alone or in combination with PhIP, whatever the cell line : 32±6 dG-N^2^-BPDE-DNA adducts for B(*a*)P alone versus 27±1 dG-N^2^-BPDE-DNA adducts for PhIP with B(*a*)P for Apc^+/+^ cells, and 45±4 dG-N^2^-BPDE-DNA adducts for B(*a*)P alone versus 63±13 dG-N^2^-BPDE-DNA adducts for PhIP with B(*a*)P for Apc^Min/+^ cells (Fig. 4A and Table 2). In contrast, the addition of B(*a*)P induced a 5-fold increase of the amount of PhIP derived DNA adducts on Apc^+/+^ cells (12±1 dG-C8-PhIP DNA adducts for PhIP alone versus 64±10 dG-C8-PhIP DNA adducts for PhIP with B(*a*)P) and on Apc^Min/+^ cells (93±20 dG-C8-PhIP DNA adducts for PhIP alone versus 468±23 dG-C8-PhIP DNA adducts for PhIP with B(*a*)P) (Fig. 4A and Table 2).

The γH2AX assay was also performed with the mixture of B(*a*)P (1 µM) and PhIP (10 µM) on the two cell lines (Fig. 4B). An increase in H2AX phosphorylation was observed for both cell lines with the mixture, but the phosphorylation levels were not significantly different from the levels observed with B(*a*)P alone (Fig. 4B and Table 2).

## Discussion

In the present work, we focused on two known carcinogens, B(*a*)P and PhIP, both relevant to the induction of colon cancer. B(*a*)P has been demonstrated to be highly mutagenic in murine small intestine and colon *in vivo* [42]. Moreover, the formation of dG-N^2^-BPDE adducts has been demonstrated in human colon and may be implicated in human colorectal carcinogenesis [43]. Similarly, *in vivo* evidence have been provided for a relationship between the amount of dG-C8-PhIP DNA adducts and the carcinogenic property of PhIP in *Apc* (min) mice [12], [44], [45].

To investigate the possible link between diet’s contamination by mutagens and the early steps of colorectal carcinogenesis *in vitro*, we used two intestinal cell lines simulating the early step of colorectal carcinogenesis with an heterozygous *Apc* genotype [14], [15], [16], [17]. Since a higher metabolization of PhIP in Apc*^Min/+^* cells with respect to Apc*^+/+^* cells was previously reported [21], an extensive comparative metabolic study of B(*a*)P was performed on these two cell lines. Two studies have previously shown that in Apc^Min/+^ cells, CYP1B1 expression is higher than in Apc^+/+^ cells [21], [22]. These studies also demonstrated an increase of CYP1A1, 1A2, and 1B1 expression in the two cell lines after treatment with TCDD, an inducer of the CYP1 family [21], [22]. For PhIP, which requires a first step bioactivation by these enzymes to form DNA adducts [18], Bellocq et al. hypothesized that more DNA adducts could be generated in Apc*^Min/+^* cells than in Apc*^+/+^* cells [20]. Like TCDD, B(*a*)P is an inducer of the CYP1 family [41]. Moreover, CYP1A1, 1A2, and 1B1 are also the main CYPs involved in B(*a*)P metabolism [19]. The increase of expression of these enzymes, which are directly involved in the bioactivation of B(*a*)P, could play a role in the highest metabolic efficiency of Apc^Min/+^ cells observed in this study for this compound. In humans, the ingestion of chargrilled meat, which is known to contain high amounts B(*a*)P, has been shown to be associated with an induction of CYP1A activities both in the liver and the small intestine [46]. These results are also in agreement with recently published data demonstrating that microsomes prepared from colon tumor of Apc^Min/+^ mice are more efficient to biotransform B(*a*)P than microsomes prepared from the non-tumor tissues [47], which fact also suggests that our intestinal cell lines are a relevant model to mimic the early step of colorectal carcinogenesis. A previous report has shown that the mutation of β-catenin up-regulates the expression of some CYP1A isoforms in mouse liver tumors [48]. According to these results, we therefore hypothesize that a disruption of the transcriptional activity of β-catenin by the mutation of *Apc* [11] in Apc^Min/+^ cells may up-regulate the expression of CYP1A, as observed when a direct mutation of β-catenin occurs.

The genotoxicity of PAHs and HCAs is known to be due to their metabolic bioactivation, leading to the formation of highly reactive metabolites [19], [20], [26]. Accordingly, we investigated whether B(*a*)P and PhIP could have a differential DNA damage impact on Apc^Min/+^ and Apc^+/+^ cell lines. As a first approach, we used a technique based on the direct quantification of derived DNA adducts produced on the two cell lines (dG-N^2^-BPDE and dG-C8-PhIP for B(*a*)P and PhIP, respectively) using HPLC-MS/MS. Then, a genotoxic assay based on H2AX phosphorylation quantification was developed and applied. The H2AX assay was already shown to be suited for the screening of PAHs genotoxicity [28], [29]. For both compounds, a clear difference between the two cell lines was found, with more dG-N^2^-BPDE DNA adducts or dG-C8-PhIP DNA adducts observed with Apc*^Min/+^* cells, compared to Apc*^+/+^* cells. Dingley et al. have observed higher PhIP DNA adduct levels in the tumor tissues compared to the normal tissue in humans [23]. Because most of the human tumors present an *APC* mutation [11], this *in vivo* work using human tumors is in good agreement with our *in vitro* results with cells mutated for *APC*. A similar differential genotoxic pattern was observed using the H2AX assay, confirming a more effective genotoxic effect of B(*a*)P and PhIP on Apc^Min/+^ than on Apc^+/+^ cells. The DNA adducts measurements as well as the level of phosphorylation of histone H2AX, were in good agreement with the metabolic capabilities, and therefore with the bioactivation differences, observed between the two cell lines tested for B(*a*)P in this study and for PhIP previously [21], [22]. These results with colon cells are also in good accordance with the fact that B(*a*)P was shown to be highly mutagenic in murine small intestine and colon *in vivo* [42], and with the *in vivo* dG-C8-PhIP DNA adducts measurement and carcinogenic property of PhIP in *Apc* (min) mice [12], [44], [45].

The observed H2AX phosphorylation in the presence of B(*a*)P or PhIP was mainly due to the replication and transcription block by derived DNA adducts [49], [50]. Interestingly, we observed that for the same number of DNA adducts (32 DNA adducts/10^8^ normal nucleosides on Apc*^+/+^* cells), the increase in H2AX phosphorylation was lower with PhIP than with B(*a*)P derived DNA adducts (1.27 and 2.18 fold increase compared to DMSO, respectively). For both molecules, DNA adducts are formed on a guanine residue, but not at the same position of guanine: N^2^ for BPDE and C8 for PhIP. Furthermore, PhIP molecule displays an out of plane geometry whereas BPDE is a planar ligand. These structural differences lead to different conformations in the double strand DNA [20], [26]. These conformational differences could reflect the property of each of these DNA adducts to block replication and transcription in different ways or to be bypass by specialized DNA polymerase. This may explain the differences of H2AX phosphorylation we measured in our experiments. Similar observations were reported in a previous study that demonstrated different DNA transcription blocking properties for three different types of DNA adducts in human cells [51].

Because B(*a*)P and PhIP can be present simultaneously in the diet and could present combined effect on mutagenesis [52], we also investigated the genotoxic effect of the mixture of these two mutagenic food contaminants in our cellular assay. We used a 10/1 PhIP/B(*a*)P ratio, considering that this proportion would correctly reflect food contamination, as deduced from different quantitative measurement [53], [54], [55], [56]. As far as we know, this is the first time that B(*a*)P and PhIP derived DNA adducts were quantified in the same study. For PhIP derived DNA adducts, a 5 fold increase of dG-C8-PhIP-DNA adducts was observed in the presence of B(*a*)P compared to PhIP treatment alone, on both cell lines. In contrast, for B(*a*)P derived DNA adducts, no significant difference was detected between dG-N^2^-BPDE-DNA adducts after treatment with B(*a*)P alone or in combination with PhIP, whatever the cell line tested. Due to the fact that the two types of DNA adducts are repaired by the same DNA reparation pathway, namely nucleotide excision repair [57], the saturation of this DNA repair pathway cannot explain the combined genotoxic effect of B(*a*)P and PhIP in our experimental conditions. The potentialization of PhIP DNA adducts formation by B(*a*)P more likely results from an increase of PhIP metabolic bioactivation, consequent to an induction of CYP1 activities by B(*a*)P [19]. The H2AX assay with the mixture of B(*a*)P and PhIP resulted in an increase in H2AX phosphorylation in both cell lines, although the response was not significantly different compared to the effect measured for B(*a*)P alone. Our results showed that a 10 fold increase in PhIP DNA adducts formation was necessary to provoke a marked raise of the H2AX signal, while a 5 fold increase in DNA adducts formation was necessary in the case of B(*a*)P DNA adducts (Table2). When exposing cells to the PhIP/B(*a*)P mixture, we observed only a 5 fold increase in PhIP DNA adducts formation compared to the PhIP alone treatment (Table 2). This increase may be insufficient to induce a significant increase of the γH2AX signal. So, we could not observed in our study the combined genotoxic potential of the PhIP/B(*a*)P mixture with the γH2AX assay, but this may be related to a lower sensitivity of this assay to detected PhIP DNA adducts compared to HPLC-MS/MS PhIP DNA adducts quantification method.

In conclusion, our study highlights the genotoxic potential of two common foods carcinogens, on intestinal cell lines, notably on heterozygous *Apc* mutated cells, which reflect early steps of colorectal carcinogenesis. A combined genotoxic effect of these two compounds was tentatively observed. From these results, it appears that PAHs may be implied in the process of colon cancer initiation, and that both AAHs and HAPs may be involved in colorectal carcinogenesis progression. This hypothesis is in agreement with many epidemiological studies that have demonstrated a link between DNA adducts repair potency and the risk of colorectal cancer [58], [59], [60]. Nonetheless, additional mechanistic studies, notably *in vivo,* must be undertaken to unequivocally confirm the combined genotoxic effects of PAHs and HCAs, as well as their potential involvement in the colon carcinogenesis process.
